# Cases of Chronic Disease—Cured*Continued from *The Organon*, vol. iv, p. 54.

**Published:** 1881-06

**Authors:** Thomas Skinner

**Affiliations:** Liverpool


### CASES OF CHRONIC DISEASE—CURED.*

THOMAS SKINNER, M.D., LIVERPOOL.

### *Cases of Displacement of the Uterus.*

Retroversion and Subinvolution of the Uterus—Calcarea.—Mrs. S., a German lady, consulted me on the 10th of October 1876, for what she called "uterine displacement." She had been

* Continued from *The Organon*, vol. iv, p. 54.

under treatment of an allopathic kind for months without any relief. The physicians attending her were men of excellent repute, and they did their very best for her. Being consulted about the "uterine displacement," I, at once, made a vaginal examination. I found the womb retroverted, the os patent, and unmistakable subinvolution of the uterus. With all deference to her previous physicians, the displacement was not all she was suffering from, as she was suffering from *Psora*, and psora was the real cause of the displacement, if I am not mistaken. I was told that the womb had been frequently rectified by means of the uterine sound, but it had returned to its retroverted condition. Nevertheless, the temptation to redress the uterus was more than a match for me; so, with the aid of the sound and the two fingers of my right hand, I replaced the organ in its natural position. I then took stock of my patient. Her age, in 1876, was thirty-eight; she was married and the mother of several children. She complained of soreness of the womb, worse when rocking the baby, or from external pressure, or from the motion of a carriage; she suffers also from leucorrhœa like white of egg and stringy, worse in the day and from carriage exercises. *The menses are regular but profuse; occasionally a day or two too soon.* They are accompanied by dark clots, and a dark flow day and night. She has a tendency to ill-humor before and during the flow, but much less than formerly. *Specks before the eyes, black.* Noises in the back of the head and ears, especially on moving the head. *She is fearfully timid and nervous. Hot flushes and warm sweats; perspires in bed toward morning, hot at first, but becoming clammy;* above all, she has a sensation *as if her stockings were damp.* On the 10th Oct., 1876, she got one dose of the *one hundred and fifty thousandth* attenuation of *calcarea carb.*, and on the 30th Oct. and 13 Nov. of the same year she reported herself perfectly well in every respect. I made no further examination.

Asthma during the Menses—Kali Carb.—On the 16th of July, 1878, the same patient, about two years after the above narrated cure was effected, called to consult me on account of nocturnal fits of asthma, with slight expectoration and cough during the menses. Her symptoms then were as follows: Difficulty of breathing, a tightness, especially about the root of the neck. As a rule, she generally *wakes with the worst bout, about* 3 A.M., and again on rising. She can not do with any thing tight round her waist. Aggravation of the asthma *during* the M. P. On the 16th July, 1878, she was directed to dissolve a few globules of *kali carb.* C. M. (F. C.), in a wine glass of water, one teaspoonful to be taken, when an attack is on, every hour till better.

COMMENT.—On the second occasion, when she called about her asthma, I asked her regarding the retroversion *et cetera*, and she replied that "she did not now know that she had a womb," she felt so well in that quarter. I made no examination; and wherefore should I, when she felt quite well? Mrs. S. "never looked over her shoulder" after the first dose of *kali carbonicum*, as the asthma has never since troubled her. Indeed, the only trouble she has had since her last visit is the death of her beloved husband, who died in other hands than mine. In the first part of this case, the symptoms corresponded strongly to *sepia*, and doubtless if *calcarea* had failed, *sepia* would have been the *Simillimum.* As it is, nothing could have acted more satisfactorily than the single dose of *calcarea* 150 M. It is evident that the redressing of the displacement did good, but without the *calcarea* it had frequently failed to be permanent even in affording relief.

PROLAPUS UTERI.—R. D. (colored) who goes out to wash and iron, was sent to me by a lady friend, who takes much interest in the woman. Her age is fifty-four and the menopause began ten years ago. She is a widow who has only had one child and three miscarriages. On the 3d April, 1878, she informed me that she had suffered on and off from falling down of the womb for *thirty years;* for the last two years she had worn a pessary, but ultimately it had to be removed on account of aggravating her misery. For the first two months it afforded relief to her symptoms. She has worn no pessary for two years.

SYMPTOMS.—She has *great pain in the left iliac region and groin, worse when walking or standing, alleviated by sitting.* She thinks she feels the womb return into its place when sitting. She has frequently *a feeling as if she would lose the use of her left leg.* She suffers from lumbar pains of a sharp shooting or *cutting character, proceeding from right to left, settling down in the left groin.* She has a dragging pain *under the left ribs in front and to the side. The left side is her weak one*, the right being altogether free. She has occasional white discharges, and *she is greatly troubled with wind.* She is *habitually and obstinately constipated. Hot flushes to face; subject to violent headaches of a throbbing character, and her feet are always cold and dry.* Can any one doubt the corresponding remedy? On the 3d of April 1878, I gave her one dose of *lycopodium* C.M. (F.C.) there and then, dry on her tongue, and she was to call in a week. She called when I was ill and in bed, so she called again on the 15th of April complaining that ever since she saw me she had experienced an aggravation of all her symptoms, *especially the pain in the left groin.* There was no doubt in my mind that it arose from the C.M. of *lycopodium*, and be it remembered that it had lasted twelve days. I gave her one dose of the M.M., and on the 23d of April, she called to say that the great bearing down and prolapsus had entirely left her, but *the pains* in the left hypochondrium and *left groin* are no better. She got *thuya* 30 (F.C.) night and morning until she felt better or worse. April 31st, 1878. She reported great benefit from the *thuya.* The pain at the bottom of the left ribs is gone, the first dose affecting it, but it returned. Great pain is felt across the small of the back, and in the left groin. She got no medicine; to return in a week. May 8th, 1878. The prolapsus uteri sensation is much less often than formerly; the bearing down is still in abeyance, and the left infra mammary and inguinal pains are almost gone. No medicine. July 17th, 1878. Return of the *lumbar pain*, the paralytic feeling in left leg, and the sensation of prolapsus uteri. *Eructations of wind affording great relief.* She got one dose of *antimonium tart.* 1600. July 24th, 1878. She reports: all her symptoms are gone again, except the pain in the lumbar region, as if broken or beaten, the weak feeling in her left leg, and an occasional sense of prolapsus. These symptoms are *worse in cold, damp weather; she is drowsy at all times during the day; distension after meals; backache and always wakens with her mouth parched without thirst.* She received *nux moschata* 500 (F.C.) July 31st, 1878. The pain in the back is greatly better, "not nearly so racking and violent." The weak feeling in the left leg is also much better, and is still improving. No medicine. August 8th, 1878. The feeling of paralysis in the left leg is entirely gone, and the backache is much less. September 17th, 1878. She feels now as if she were "perfectly well; better than ever she has felt in her life." One month after this (17th, Oct.) she felt a return of the prolapsus, with an aching across the small of her back, worse in bed of a morning; she had also a red discharge with cutting pains in the hypogastrium and loins *aggravated in wet weather, and full of wind.* I gave her *carbo vegetabilis* 50 M. (F.C). one dose. October 31st, 1878. Greatly better in all respects, and improving in health and strength. November 14th 1878. She called to return thanks, and to say that she has not felt so well for years, and that she is now able to do her work with comfort. I discharged her as cured, and that if ever there was a return of her symptoms she was to call or inform her friend, Mrs. D. As she has done neither, I feel justified in concluding that she remains well, if in the land of the living.

COMMENT. — The only comments which I think necessary to make are (1). This was a genuine case of *prolapsus uteri*, as I examined her and found the neck of the womb swollen and protruded fully two inches beyond the vulva. The womb was rarely more prolapsed, but she often felt as if the whole of her insides, womb and all, would come out. I did not examine her when she left me, because the bearing down was gone, which is the disease. The bearing down is the cause, the prolapsus the effect. Remove the cause, and the effect, the prolapsus, ceases. (2). I have no doubt, that, if I had not been in such a hurry to change the first *simillimum* and had only stuck to it throughout, I should have accomplished the cure more quickly, much more homœopathically, and altogether much more satisfactorily so far as my own feelings are concerned. In conclusion, here is a case of *Prolapsus uteri* of *thirty years* standing in a washerwoman, cured in about *seven months*, without mechanical support of any kind, without local treatment, and what is most extraordinary, while she was following her laborious vocation, mostly standing. When the advocates of local treatment can show us equal or superior success, it will be time enough for them to find fault, or to ask us for proof of our ability to do away entirely with local medication. No one can doubt, that if this poor woman could have obtained rest and the necessities of life without standing and working for them, and having to go out in all weathers, a speedier cure might have resulted; although I think that seven months is a short enough time to subdue thirty years of psoric misery.

